# CGHScan: finding variable regions using high-density microarray comparative genomic hybridization data

**DOI:** 10.1186/1471-2164-7-91

**Published:** 2006-04-25

**Authors:** Bradley D Anderson, Michael C Gilson, Abigail A Scott, Bryan S Biehl, Jeremy D Glasner, Gireesh Rajashekara, Gary A Splitter, Nicole T Perna

**Affiliations:** 1 Animal H ealth and Biomedical Sciences, University of Wisconsin, Madison WI 53706, USA; 2 Genome Center of Wisconsin, University of Wisconsin, Madison WI 53706, USA

## Abstract

**Background:**

Comparative genomic hybridization can rapidly identify chromosomal regions that vary between organisms and tissues. This technique has been applied to detecting differences between normal and cancerous tissues in eukaryotes as well as genomic variability in microbial strains and species. The density of oligonucleotide probes available on current microarray platforms is particularly well-suited for comparisons of organisms with smaller genomes like bacteria and yeast where an entire genome can be assayed on a single microarray with high resolution. Available methods for analyzing these experiments typically confine analyses to data from pre-defined annotated genome features, such as entire genes. Many of these methods are ill suited for datasets with the number of measurements typical of high-density microarrays.

**Results:**

We present an algorithm for analyzing microarray hybridization data to aid identification of regions that vary between an unsequenced genome and a sequenced reference genome. The program, CGHScan, uses an iterative random walk approach integrating multi-layered significance testing to detect these regions from comparative genomic hybridization data. The algorithm tolerates a high level of noise in measurements of individual probe intensities and is relatively insensitive to the choice of method for normalizing probe intensity values and identifying probes that differ between samples. When applied to comparative genomic hybridization data from a published experiment, CGHScan identified eight of nine known deletions in a *Brucella ovis *strain as compared to *Brucella melitensis*. The same result was obtained using two different normalization methods and two different scores to classify data for individual probes as representing conserved or variable genomic regions. The undetected region is a small (58 base pair) deletion that is below the resolution of CGHScan given the array design employed in the study.

**Conclusion:**

CGHScan is an effective tool for analyzing comparative genomic hybridization data from high-density microarrays. The algorithm is capable of accurately identifying known variable regions and is tolerant of high noise and varying methods of data preprocessing. Statistical analysis is used to define each variable region providing a robust and reliable method for rapid identification of genomic differences independent of annotated gene boundaries.

## Background

Comparative genomic hybridization (CGH) is a powerful technique to determine the differences between the genomes of different cell types or organisms. Typically, genomic DNA from known (sequenced) and experimental (unsequenced) genomes is labeled and hybridized to an array of DNA sequences prepared from the known genome. Intensities of hybridization of the two samples are compared to determine the relative copy number of each target gene in the experimental genome as compared to the known genome. Many studies involve determining the genomic abnormalities in tumor cells, but the technique has also been used in bacteria [1-6] and yeast [7-9] to investigate diverse topics such as genotyping, pathogenicity, and microbial evolution. CGH experiments on cancerous and non-cancerous tissues are primarily used in determining the relative copy number of sequences in the tumor cells as compared to the normal tissue for the purpose of identifying genetic elements responsible for cell transformation. To provide wide coverage of relatively large genomes such as human on a small number of arrays requires using large genomic fragments such as bacterial artificial chromosomes (BACs), or by using a large number of arrays [10]. Experiments in yeast and bacteria can typically be conducted at much higher resolution due to their smaller genomes, although comparisons must be limited to closely related species, as evolutionary divergence will affect hybridization dynamics if the species are significantly diverged. A single spotted cDNA or oligonucleotide array can easily represent every open reading frame (ORF) in the genome, and a high-density microarray can provide sequences that cover the entire genome. Typical high-density microarrays contain hundreds of thousands of short oligonucleotide (~25 nucleotides) sequences (probes). The substantial variability in performance of different probe sequences and the large number of individual measurements taken complicate the analysis of hybridization data. Many methods have been developed for analyzing data from human array CGH using cDNA or BAC arrays [11-18].

A common theme emerging from comparisons of closely related bacteria is genomic variability due to insertion and deletion of genes and gene islands. Regions that are present in a sequenced genome and absent or significantly diverged in an experimental genome can be readily detected by hybridization. We have termed these "variable regions" because based on CGH alone, it is not possible to distinguish between deletions and sequences that have diverged to a degree that prevents or reduces hybridization. Defining the boundaries of these regions from high-density microarray data involves scoring each probe as indicative of either a conserved or variable sequence and grouping probes with consistent scores into larger segments with defined boundaries. While there are many methods of scoring probes [15,18-21], boundary definition remains a challenge. A simple approach to detect deleted genes is to average probe values across each gene and select genes with unusually low relative hybridization values [3]. This method defines the units-of-interest as entire genes and does not attempt to identify variable regions that lie outside of genes or only partially span gene boundaries. Larger clusters of variable genomic regions are defined by grouping adjacent variable genes, but definition of the boundaries of these clusters can be sensitive to inclusion or exclusion of individual genes in the initial analysis. An approach developed for bacterial CGH, implemented in a program called TSTEP, identifies deletions using microarray data by scoring probes as absent or present based on perfect match and mismatched probe intensities as well as consideration of neighboring probe data using a sliding window [20]. A heuristic set of rules is then applied to find clusters of absent probes. Other programs, developed to analyze mammalian CGH data, analyze data by smoothing probe intensities, followed by breakpoint identification by scanning for areas of high contrast between smoothed data values of neighboring probes [12-15,18].

In our efforts to analyze CGH data from high-density microarrays, we found several common drawbacks with existing methods. Many methods simply score probes or genes as present or absent but do not provide a mechanism for defining region boundaries. Most of the available software for defining boundaries was developed for analyzing data from human spotted microarrays, and we encountered problems such as excessive setup and/or run time, dependence on genome annotation, input file format restrictions, and an inability to handle large data sets. The data we used to develop our approach come from a high-density array with nearly 200,000 individual 24 mer probes, whereas the human data comes primarily from spotted arrays with far fewer probes of longer length. The larger size of our dataset and greater variability inherent in the performance of short probe sequences compared to longer probes precludes the use of most existing approaches. For these reasons, we developed CGHScan, a program that uses an iterative random walk method to detect variable regions using high-density CGH microarray data. The algorithm identifies variable regions in data with high levels of noise independently of genome annotation, providing a rapid method for defining differences in high-density microarray CGH studies.

## Implementation

CGHScan operates on a data set *D *comprised of binary data wherein each probe has been scored either conserved or variable in the experimental genome. The data is input as a tab delimited text file containing the genomic coordinate of each probe along with which chromosome it corresponds to and the data value. Use of a proprietary file format was done to make the program usable irrespective of the software used by the experimenter to analyze their microarrays. We score conserved probes as 1 and variable probes as -1, but CGHScan can operate on any binary numerical scoring method, providing that the score of the variable probes is less than the score of the conserved probes. We strongly advise users to select a statistically rigorous method to score individual probes such as GACK or EBArrays [21,22]; However, if the data supplied by the user is in the form of raw numerical values for each probe CGHScan will perform a simple scoring based on a user-defined cutoff value. Since the grouping of probes into variable genomic regions relies on the scores of individual probes, the choice of an appropriate method for initial scoring of probes is important and it is advisable to try using different scoring thresholds to confirm that the results are robust for a given data set. CGHScan requires the relative physical location of each probe sequence in the reference genome.

### The random walk as a method for boundary definition

While many methods of analyzing binary data have been described [23-25], we chose the random walk. A one-dimensional random walk occurs on a line, where a series of equidistant steps are taken in one of the two available directions. The probability of taking a step down is defined as *P*, while *Q *is the probability of taking a step up. A random walk of *N *steps is necessarily comprised of a number of down steps, *n*_*d *_and up steps, *n*_*u *_such that *n*_*d *_+ *n*_*u *_= *N*. The probability of any single random walk occurring is given by the equation

(nd+nu)!nd!nu!PndQnu
 MathType@MTEF@5@5@+=feaafiart1ev1aaatCvAUfKttLearuWrP9MDH5MBPbIqV92AaeXatLxBI9gBaebbnrfifHhDYfgasaacH8akY=wiFfYdH8Gipec8Eeeu0xXdbba9frFj0=OqFfea0dXdd9vqai=hGuQ8kuc9pgc9s8qqaq=dirpe0xb9q8qiLsFr0=vr0=vr0dc8meaabaqaciaacaGaaeqabaqabeGadaaakeaadaWcaaqaamaabmaabaGaemOBa42aaSbaaSqaaiabdsgaKbqabaGccqGHRaWkcqWGUbGBdaWgaaWcbaGaemyDauhabeaaaOGaayjkaiaawMcaaiabcgcaHaqaaiabd6gaUnaaBaaaleaacqWGKbazaeqaaOGaeiyiaeIaemOBa42aaSbaaSqaaiabdwha1bqabaGccqGGHaqiaaGaemiuaa1aaWbaaSqabeaacqWGUbGBdaWgaaadbaGaemizaqgabeaaaaGccqWGrbqudaahaaWcbeqaaiabd6gaUnaaBaaameaacqWG1bqDaeqaaaaaaaa@4619@

which is the binomial probability.

**Figure 1:**
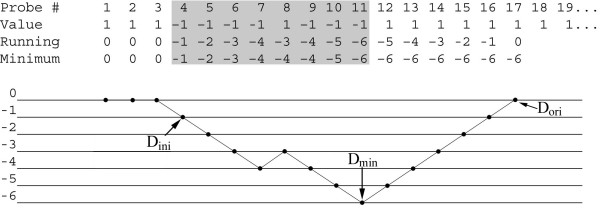
An illustration of the random walk. A region is defined by D_ini _and D_min_, shown here as a shaded area spanning probes 4–11.

In order to define the boundaries of variable regions, the genome-ordered probes are scanned beginning with probe *D*_1 _until a variable probe is encountered, probe *D*_*init*_, and a one-dimensional random walk begins, with conserved and variable probes contributing +1 (step up) and -1 (step down) respectively. Assuming that the genomes being compared consist largely of conserved regions, the walk (score) will eventually return to the origin at probe *D*_*ori*_, and have a minimum at probe *D*_*min *_(Fig. 1). In the event of multiple identical minima, the *D*_*min *_closest to *D*_*ori *_is defined as the location of the minimum. If the walk does not reach the origin before the end of the chromosome, the last probe in the chromosome is defined as *D*_*ori*_. The boundaries of the variable region are defined by *D*_*init *_on the left side and *D*_*min *_on the right, as this represents the longest region of local concentration of variable probes. The coordinates are recorded as a region and the scan resumes at probe *D*_*min *_+1, where, once again, all conserved probes are ignored until the first variable probe is encountered, and another random walk is initiated. This process is repeated until the end of the genome is reached. Analyses on circular genomes where there is the possibility that D_1 _lays within a variable region should be re-analyzed with an alternate D_1 _to ensure proper analysis of the region. Multiple chromosomes of a genome are scanned separately and in parallel.

**Figure 2:**
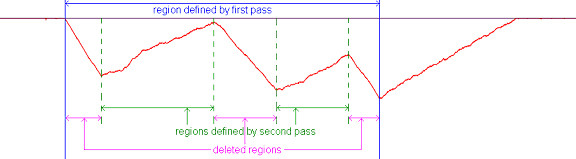
The second iteration detects conserved regions within first iteration regions.

A second iteration scans for conserved regions within the regions found in the first iteration. This is necessary because the random walk tends to combine variable regions separated by a relatively small conserved region. The requirement for ∑(*D*_*init*_,...*D*_*ori*_) assumes that each variable region will be followed by a sufficiently long stretch of conserved probes. When two regions are close together, they can be grouped together by the random walk (Fig. 2). Solving this problem requires an automated method for identifying significant local minima, while ignoring minor local minima caused by noise. This can be approached in two different ways. One possible solution is to record the most recent minimum *D*_*m*1_, and as the walk proceeds calculate the statistical significance of the regions defined by *D*_*m*1_+1 and the current probe. Using this method, the walk does not need to return to the origin, but merely significantly far away from the minimum providing a new minimum is not established. The alternative solution, employed in CGHScan, scans each region identified in the first iteration for conserved regions, using the same random walk method. Using this approach we can statistically evaluate the identified conserved regions as a group and are able to employ a correction for multiple tests. Regions found in this second iteration are similarly scanned for variable regions in a third iteration, which are scanned in for conserved regions in a fourth iteration, and further iterations are performed until no additional regions are detected.

### Statistical analysis of regions

For an operation of *N *iterations, probabilities are calculated for regions defined in the *N*^*th *^iteration first. As these regions are detected as lying within regions defined in the previous iteration, *P *and *Q *are defined as the proportion of variable and conserved probes, respectively, that comprise the regions identified in the previous iteration. Regions defined by a random walk from *D*_*init *_to *D*_*min *_can be statistically evaluated using the binomial distribution. For smaller regions, the binomial probabilities can be calculated directly. While the binomial probability is a useful metric for the user to evaluate regions, all evaluation of these regions for statistical significance are performed using the method developed by Tarone [26] which employs the minimum possible probability *P*^*n*^, which is described below. For larger regions, we use an approximation of the binomial probability. Larger regions are defined as those where the number of probes multiplied by the smaller of *P *or *Q *is greater than or equal to 5, and binomial probabilities are approximated using the z-test. For regions where the binomial probability is approximated with a z-test, a Bonferroni multiple testing correction is used. For the smaller regions, a modified Bonferroni correction described by Tarone [26] developed specifically for discrete data is used. Briefly, for any region with a length of n probes, P^*n *^represents the minimum possible binomial probability for the region. Beginning with an integer *k *= 1, the regions that satisfy the criterion k*P^*n *^< α comprise a subset *N(k) *of all total regions *N *in a given iteration. Integer k is increased by one until *k ≥ N(k)*. This modification removes regions from consideration that could not reject the null hypothesis under any circumstances and makes the correction less conservative. A standard Bonferroni correction is applied to the remaining regions using the minimum binomial probability (*P*^*n*^) for each region. Regions that are called significant after the multiple correction tests are then "added back in" to the regions in the previous iteration in which they were found. For example, a putative variable region from iteration *N*-1 spanning probes *D*_*a *_to *D*_*z *_containing a statistically significant conserved region from iteration *N *spanning probes *D*_*x *_to *D*_*y *_will be broken into two regions, one spanning *D*_*a *_to *D*_*x*-1_, the other spanning *D*_*y*+1 _to *D*_*z*_. This process is repeated with iteration *N*-1 and so on until iteration 1 is reached, where regions are evaluated against *P *and *Q *defined as the proportion of variable and conserved probes in the genome, respectively.

### Output

The output is a table reporting the genomic coordinates of all predicted variable regions with their binomial probability or approximation. Two additional tables are output, one detailing all larger variable regions that were identified using the z test, and another for the smaller variable regions analyzed by the modified Bonferroni correction. In the last two tables, variable regions are reported and are grouped by the iteration that they were identified in regardless of whether they passed the Bonferroni correction to permit users to evaluate regions near the cutoff.

## Results

CGHScan was used to analyze microarray data from a study by Rajashekara et al. [3] comparing the sequenced pathogen *Brucella melitensis *strain 16 M with the closely related *Brucella ovis *strain REO198. The 3.3 Mbp *B. melitensis *genome is contained on two chromosomes. The microarray contains 17 probes per open reading frame, plus a number of probes corresponding to intergenic regions, which combined provide extensive, albeit partial, coverage across the entire genome. The trimmed mean of the middle 50% of ordered probe intensities was used to calculate a scaling factor to normalize probe intensities from three replicate experiments for each strain, which were then averaged. Log2 ratios of the average probe intensities between the experimental and known genome were calculated and input into CGHScan using a significance value of 0.05. We tried using two different log2 ratio thresholds calculated by GACK, a program designed to identify probes with extreme ratio values from microarray data [21]. The first threshold, -0.319, was calculated using the most lenient (100%) GACK setting for creating binary (scored) data, and the second, -0.619, using the default setting (50%), which results in fewer probes being classified as variable.

**Figure 3:**
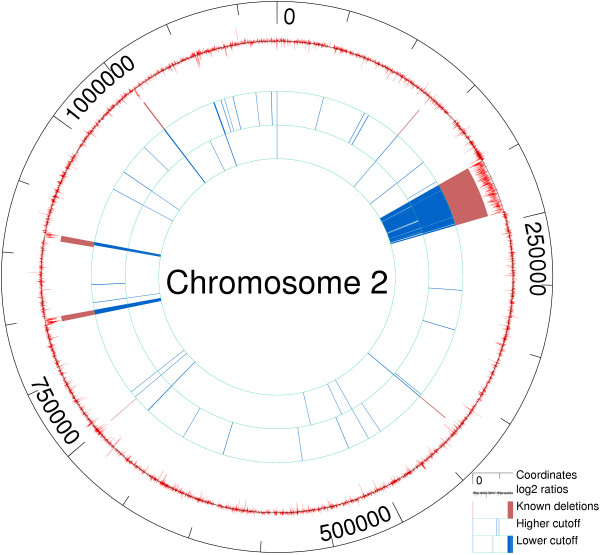
Deletions and variable regions in *B. melitensis *chromosome II. Regions in red are from Rajashekara et al., (2004). Blue regions are predicted by CGHScan, outer ring using a higher cutoff (which results in lower stringency), and the inner ring with a lower cutoff (which results in higher stringency).

*B. ovis *contains nine known deleted regions confirmed by sequencing as compared to *B. melitensis *[3]. CGHScan identified eight of the nine regions at both ratio thresholds, two in chromosome I, six in chromosome II (Fig. 3). CGHScan successfully identified these eight regions with a ratio threshold as low as -0.820, indicating that the algorithm is effective over a range of ratio thresholds. The only previously known deletion that CGHScan did not identify is a 58 bp deletion that was not detectable using our approach given the probe density of the microarray. Some of the larger known deletions were identified by CGHScan as a cluster of smaller variable regions, particularly with the lower ratio threshold (Fig. 3). This is partially due to the fact that the control strain contains known duplications of portions of these regions, which results in higher log2 ratios than expected in those areas. The use of the lower ratio threshold can cause regions to become segmented due to the fact that more probes are called conserved, but segmented regions are in our experience easily recognizable as a single entity (see Fig. 3). In addition to identifying eight of the nine known deletions, many additional putative variable regions were identified using either cutoff, with more regions being detected using the less stringent cutoff. One of these regions contains a repeat present in multiple copies in 16 M, but apparently single or at least fewer copies in REO198, causing reduced sequence dosage and apparent variation (unpublished observations). Most of the putative variable regions had not been previously identified. The analysis was repeated using data normalized against the mean instead of a trimmed mean to test performance using an alternative normalization method. GACK was used to establish the default cutoff and a low-stringency cutoff. Once again, CGHScan identified the same eight known deletions, as well as other putative variable regions, with more overall regions being detected using the less stringent cutoff (data not shown). Using the more stringent cutoff, the regions detected using data normalized against the mean vs. a trimmed mean were essentially identical, with only one region differing out of about 35. Using the less stringent cutoff, the differences were mostly in agreement as well, but did show more variation than at the more stringent cutoff.

CGHScan has advantages over existing software, primarily because it was specifically designed to identify clusters of variable probes from datasets consisting of hundreds of thousands of individual probe measurements from high-density arrays. Other tools accomplish similar analyses, but typically assume that the data will consist at most tens of thousands of measurements from a single array, such as data generated from spotted arrays or from high-density arrays with data condensed into genic units. For example, CGH-miner version 1.0, which implements the "cluster along chromosomes" or CLAC method [15], and aCGH-Smooth version 1.0.0.1 [14] require data input as Microsoft Excel files, which cannot accommodate the large file sizes generated by high-density microarray experiments. We encountered errors using data sets containing as low as 16,000 probes using CGH segmentation [16], and after hours it was unable to complete the analysis of a data set consisting of 8,000 probes, less than one tenth that of a typical high-density microarray data set. CGHPRO [18] is a stand-alone java application that provides a comprehensive environment for analyzing CGH data that includes data normalization, analysis and visualization components. The software, however, requires a MySQL database and the statistical program R, making installation difficult for many potential users without the expertise necessary to install the multiple required components. CGH-Explorer [11] is configured to analyze only data from human arrays and we were unable to reconfigure it to use another genome.

In order to assess the performance of existing applications on high-density microarray data sets and compare their results to CGHScan it was often necessary to use only a portion of the data set, otherwise many programs would not be able to accommodate the data or complete the analysis. Applications with the option to define parameters were all run at their default settings. As previously mentioned, using CGH segmentation [16] we were unable to obtain results using a data set of as few as 8,000 probes, which itself was too small a portion of the data to do a meaningful comparison. ChARM version 1.6 [13] was the only program that we tested that was able to analyze our entire data set. Using the default settings in ChARM, the program was able to identify five of the nine known deletions in *B. ovis*, with one of the regions missed being a large deletion in chromosome 1. It should be noted that the default settings for ChARM do appear to be more conservative than the settings we used to test CGHScan. Our ability to test multiple different settings using ChARM was complicated by excessive runtime, presumably due to the large sizes of our data files. ChARM required hours to analyze a single data set, compared to seconds for CGHScan, and ChARM was frequently unable to complete the analyses. aCGH-Smooth version 1.0.0.1 [14] limits its input to Microsoft Excel files which have a limit of 65,536 rows of data. We found that in practice using data sets of greater than 30,000 probes in aCGH-Smooth caused errors that did not permit completion of the analysis. We ran a data set consisting of 28,508 probes from chromosome 2 that contained all seven of the known deletions on that chromosome. aCGH-Smooth identified four of the seven regions, failing to detect the three smallest regions. We had similar results when using the R package GLAD version 1.4.0. GLAD was also unable to do a complete analysis on our entire data set, but was capable of analyzing the entire chromosome 2 data set of 32,882 probes. GLAD identified the three largest regions, but failed to detect the four smaller regions. CGHScan was able to identify six regions, but also missed the smallest region, a 58 bp deletion that proved to be undetectable by any method.

In general, the existing programs worked well on sample data when available, and implement useful algorithms for the analysis of human array CGH data. The programs are not, however, well suited to high-density microarray CGH data analysis, as many could not accommodate large file sizes or operate in a reasonable amount of time. Additionally, many programs available require genome annotation and analyze gains and losses on a gene-by-gene basis. High-density microarrays can measure differences on a much a smaller scale, and can even identify breakpoints that occur within genes or between genes that are refractory to gene-by-gene approaches. These difficulties led us to the conclusion that we needed to develop CGHScan to address the specific need we had for high-density microarray CGH data analysis.

## Conclusion

We have developed a robust and efficient method for detecting variable regions between genomes using comparative genomic hybridization data from high-density microarrays. The method is relatively insensitive to data normalization methods and cutoff selection and uses rigorous statistical analysis. The method successfully identified all eight detectable deleted regions identified in a published comparison of *B. ovis *as compared to *B. melitensis*, as well as numerous additional regions that are predicted to differ between the genomes of these organisms.

## Availability and requirements

Project Name: CGHScan

Project Home Page: 

Operating System: Platform Independent

Programming Language: Java

Other Requirements: Java Runtime Environment 1.4.2

License: GNU General Public License

Use: Free, no license required

## Abbreviations

CGH – comparative genomic hybridization, ORF – open reading frame

## Authors' contributions

BA co-conceived the algorithm, was the primary author of the software, drafted the manuscript, and assisted in the data and statistical analyses.

MG assisted in authoring the software, designed the graphical user interface, and was responsible for making the software distributable.

AS was responsible for the statistical analysis, and assisted in drafting the manuscript.

BB assisted in the development of the software and data analysis.

JG assisted in the data analysis, offered technical expertise, and assisted in drafting the manuscript.

GR assisted in the data analysis.

GS assisted in the data analysis.

NP conceived of the study, co-conceived the algorithm, assisted in the statistical analysis, and provided technical expertise.
